# Headaches in Moria: a reflection on mental healthcare in the refugee camp population of Lesbos

**DOI:** 10.1192/bji.2019.2

**Published:** 2019-11

**Authors:** Tom Nutting

**Affiliations:** Avon and Wiltshire Mental Health Partnership NHS Trust, General Psychiatry, Bristol, UK. Email: thomas.nutting@nhs.net

**Keywords:** Anxiety disorders, depressive disorders, human rights, post-traumatic stress disorder, social deprivation

## Abstract

Having returned from a period of volunteering with a healthcare charity working with the refugee camp population of Lesbos in Greece, a junior doctor reflects on the common presentations he saw and the current state of mental healthcare for these patients. The placement of already-traumatised people in an overcrowded and under-resourced camp environment creates a perfect storm for the emergence of post-traumatic stress disorder, depression and anxiety. With extremely limited psychiatric care in place, he considers the simple interventions he could use to help his patients with their distressing symptoms. This prompts exploration of the importance of giving time to listen as well as encouraging small but significant lifestyle changes. After exploring the ethics of psychiatric diagnosis in this setting, the author concludes that while we must acknowledge the political origins of some of the symptomatology in this population, we must continue to strive to treat psychiatric illness with all the appropriate interventions available to us in order to help those in this patient group recover and move forward.

‘Docteur, aidez-moi s'il vous plaît, j'ai un mal de tête, très mauvais, tout le temps oui, voyez ici où ils m'ont frappé, où ils m'ont torturé, ma vision aussi est mauvaise, et non je ne peux pas dormir, pas du tout…’

Twenty-six-year-old Destin had arrived four months prior to asking for my help, having taken the notorious journey across the Aegean by dinghy, fleeing the Democratic Republic of Congo several months earlier. I was now seeing him in our small flakeboard clinic, volunteering with one of the few healthcare providers for refugees in the camps of Lesbos, Greece. Listening to his pleas, I was aware of my own tension headache, which seemed to be developing almost daily now, whether as some sort of somatic countertransference or just from the sheer stress of the work. While writing up his notes, I reminded myself I owed it to Destin to try the same relaxation breathing techniques I went through with him and others to help with their somatic pains before I reached for my own paracetamol – a simple analgesic to which many of them did not have easy access. If they were lucky enough to get a number at our clinic, we could give patients five days' worth of paracetamol maximum out of our small pharmacy cupboard, the contents of which varied by donation. As to psychotropics, we did not officially dispense any but had a limited emergency stash of anxiolytics and antipsychotics for the absolute worst cases. Furthermore, if we wanted to prescribe any long-term medications, we were mostly unable to do so, because this was an extraordinarily convoluted bureaucratic process and also cost more than most could afford. Apart from paracetamol, then, we could give information on a stress-relief class run by another non-governmental organisation (NGO) and, if patients were really lucky, they could get a psychology referral for several months' time, though these were limited and inconsistent. During my stay, our provision of mental healthcare was hindered further still when a large health NGO stopped taking psychiatric referrals as they were simply too overwhelmed.

It is undeniable that a mental health disaster is emerging in Moria camp – the epicentre of the refugee crisis in Lesbos, Greece, perhaps even the whole of Europe. With a nominal capacity of around 3000 residents, the camp population breached 10 000 during my stay. Though slowed over the past year or so, boats continue to arrive and spike during conflicts like the recent attacks in Syria. What has made the situation worse, however, is the EU-Turkey deal of 2016, which led to very few being moved on to the mainland. Despite this blockade, infrastructure has barely been improved in the camp, meaning there is woefully inadequate shelter, with the camp now unofficially sprawling into surrounding olive groves and children sleeping on the ground outside. Water, sanitation and hygiene facilities, too, are appalling, with approximately 1 toilet to 80 people and very poor access to showers and clothes-washing facilities. On average, people queue to get their food for around 4 h, three times a day, and what they get is nutritionally poor. Unsurprisingly, violence often breaks out in the queue for food, where tensions run high, and the highly militarised environment often reminds people of the traumas from which they fled; I saw several patients who were either injured by others in the bustling food queue or had self-injured here as they experienced feelings of panic and distress. Similarly, many of our unexplained fits and falls occurred in the food queue, as well as other post-traumatic stress disorder (PTSD) symptoms.

In terms of accessing healthcare, availability is shockingly inadequate: during my stay, the lead doctor for the only Greek governmental healthcare organisation resigned, leaving his colleague as the only non-charitable doctor for the whole camp! At this time, *Médecins Sans Frontières* psychiatrist Alessandro Barbierio wrote an open letter in which he denounced the state of Moria:

‘In all of my years of medical practice, I have never witnessed such overwhelming numbers of people suffering from serious mental health conditions, as I am witnessing now amongst refugees on the island of Lesbos. The vast majority of people I see are presenting with psychotic symptoms, suicidal thoughts – even attempts at suicide – and are confused. Many are unable to meet even their most basic everyday functions’ (MSF, 2018).

Dr Barbierio went on to articulate how the ‘island prison’ forces the already-traumatised people residing in the camp to ‘live in a context that promotes frequent violence in all its forms’, which then, of course, ‘serves as a recurrent trigger for the development of severe psychiatric symptoms’ (MSF, 2018). Out of the people the NGO International Rescue Committee see in their limited mental health programme, 41% report PTSD symptoms, 64% depressive symptoms, 55% anxiety, 60% suicidal ideation and 29% suicide attempts (IRC, 2018). The BBC reported that children as young as 10 have attempted suicide in Moria (Nye, 2018).

Although I have had some experience in refugee healthcare, both in the UK and abroad on my student elective, this was my first time working as a doctor in what is really a humanitarian crisis. What could I do to help patients when they came to me with such symptoms, distressed about their past, present and future? Although of course I would have preferred more management options, in this setting I really felt the most important thing I did was listen. Indeed, just being able to see a healthcare professional can have its own therapeutic value, especially if access is difficult, and if you can set aside some time to just listen; it felt like many of my patients had not been able to speak to anyone about the things they were speaking to me about, whether the shameful terror of auditory hallucinations, or intimate and horrifying memories of rape, or a constant fear for a child's well-being or simply quiet fury at no longer being able to study or work. The feedback we got from patients and interpreters was that our service was different because people felt trusted: they felt they were treated with respect and openness as any patient ought to be. A large part of this was time, which was a luxury – as well as an ethical dilemma: do we see more but give less? Although I set out being cautious of cross-cultural diagnoses, I found PTSD was very characteristically there in many of my patients, and it worked as a useful model to explore their symptoms with them. Psychoeducation as a treatment for PTSD has ambivalent evidence (Sin *et al*, 2017), but for us it seemed to be really helpful; many patients had not heard of PTSD, so discussing it as a common consequence of trauma really helped to validate and normalise their experiences – ‘so I'm not going mad?’, suggested Destin.

Another powerful potential therapeutic intervention lay in our clinic's placement within a flourishing community centre outside of the camp. Situated atop a breezy olive grove, the NGO – described by some as the heaven to Moria's hell – has many facilities in addition to our clinic: a school for children and adults, a women's centre, a sports centre, a gym, a legal advice centre, an allotment and a playground, as well as good food, toilets and Wi-Fi. This space and what's on offer here could act as a potent psychosocial intervention. It was surprising just how much some patients' mental states improved after a few weeks' visiting the centre, be it starting an English course or doing yoga at the women's centre. I think often just getting out of the camp was incredibly powerful in helping people feel more human and respected, and helping them hope for a better life. Several people explained how somehow this community helped them to move out of the limbo of their refugee status, which may have been a perpetuating factor in their distress and PTSD symptoms. Indeed, there is evidence to suggest that overall well-being is improved in refugees (both with and without PTSD) by building ‘resilience capital’ through access to contacts who can help them navigate the asylum process to language learning, to healthcare and health education, to exercise, and to proactive community volunteering (Melamed *et al*, 2018). Although I was not aware of this evidence at the time, I gladly clung to engagement with the community centre as one of the few suggestions I had for my patients. Indeed, its availability paradoxically prompted me to reflect upon how sometimes we might reach for medications or even psychotherapies too quickly. Don't get me wrong, it is outrageous that these are barely available to my patients in the camp – but the absence of treatment highlighted how small but significant changes to a person's day-to-day life can sometimes dramatically improve their well-being, even in the worst situations.

The lack of usual treatment options also prompted – for me – a nosological reconsideration on what makes a disease in the absence of treatment, and then what makes a disease in the presence of politics. Sometimes, too, I questioned the ethics of diagnosis in this setting, at times wondering whether it was right to medicalise the symptoms people were presenting with or to medicate away their distress; I felt this might discount their horrendous experiences and ongoing maltreatment. Does diagnosing PTSD or generalised anxiety disorder distract from the fact that this distress is in part a consequence of how these people have been treated, whether air-strikes at home, enforced sex-work as a trafficking deal, poor facilities and healthcare in the camp, or the near-constant threat of violence? Although I feel I must tell of how much mental illness there is in the refugee population in Lesbos and why it is happening and how obscenely little is being done about it, I must also ask that we do not forget how this illness burden is in part geopolitical; these people are suffering the consequences of political decisions. At the same time, however, I know that I was unable to alter what had happened to the people who came to see me, just as now I can do little to alter the circumstances of those patients who have suffered from austerity or any number of social ills in the UK. What I should be able to do, though, as ought all psychiatrists everywhere, is to bring relief – when symptoms warrant it – through all effective psychosocial and biological interventions possible in order to help people recover and to move forward.
